# Synthesis of Hydroxypropyltrimethyl Ammonium Chitosan Derivatives Bearing Thioctate and the Potential for Antioxidant Application

**DOI:** 10.3390/molecules27092682

**Published:** 2022-04-21

**Authors:** Wenqiang Tan, Conghao Lin, Jingjing Zhang, Qing Li, Zhanyong Guo

**Affiliations:** 1 Research and Development Center for Efficient Utilization of Coastal Bioresources, Yantai Institute of Coastal Zone Research, Chinese Academy of Sciences, Yantai 264003, China; wqtan@yic.ac.cn (W.T.); jingjingzhang@yic.ac.cn (J.Z.); qli@yic.ac.cn (Q.L.); 2 Center for Ocean Mega-Science, Chinese Academy of Sciences, Qingdao 266071, China; 3 College of Life Sciences, Yantai University, Yantai 264005, China; conghaolin@s.ytu.edu.cn

**Keywords:** hydroxypropyltrimethyl ammonium chitosan derivatives, α-lipoic acid, antioxidant activity

## Abstract

Hydroxypropyltrimethyl ammonium chloride chitosan (HACC) is one of the most important water-soluble chitosan derivatives; its derivatives have gained growing attention due to their potential biomedical applications. Here, hydroxypropyltrimethyl ammonium chitosan derivatives bearing thioctate (HACTs), with different degrees of substitution of thioctate, were prepared using HACC and α-lipoic acid as the reaction precursors, using an ion exchange method. The structural characteristics of the synthesized derivatives were confirmed by FTIR, ^1^H NMR, and ^13^C NMR spectroscopy. In addition, their antioxidant behaviors were also investigated in vitro by the assays of reducing power, and scavenging activities against hydroxyl radicals and DPPH radicals. The antioxidant assay indicated that HACTs displayed strong antioxidant activity compared with HACC, especially in terms of reducing power. Besides, the antioxidant activities of the prepared products were further enhanced with the increase in the test concentration and the degrees of substitution of thioctate. At the maximum test concentration of 1.60 mg/mL, the absorbance value at 700 nm of HACTs, under the test conditions, was 4.346 ± 0.296, while the absorbance value of HACC was 0.041 ± 0.007. The aforementioned results support the use of HACTs as antioxidant biomaterials in food and the biomedical field.

## 1. Introduction

The oxidative stress damage caused by the excessive accumulation of reactive oxygen species (ROS) is closely related to human aging and many neurodegenerative diseases, such as Parkinson’s and Alzheimer’s diseases, coronary heart disease, skin inflammation, carcinogenesis, and atherosclerosis [1,2,3,4]. As an effective treatment method, the supplementation of exogenous antioxidants can maintain physiological redox homeostasis and protect organisms from oxidative stress damage by scavenging the excessive ROS [5]. There is great interest in developing effective bio-macromolecular antioxidants to combat the effects of ROS [6,7,8]. Recently, some natural polysaccharides and their derivatives have been attracting attention to become promising candidates for novel antioxidants [9,10].

As one of the most versatile biomaterials, chitosan is a linear cationic polysaccharide composed of randomly distributed β-(1–4)-linked D-glucosamine and *N*-acetyl-D-glucosamine, and it was obtained from the deacetylation of chitin, which was mainly found in the exoskeletons of shrimps and crabs [11]. Chitosan has attracted much attention in the exploration as a potent antioxidant used in biomedical materials, biomedicine, and food science, mainly because of its biodegradability, biocompatibility, non-toxicity, hemostasis, healing effect, and excellent processability [12,13,14]. However, the poor water solubility and weak antioxidant action of chitosan are the major obstacles to achieve the practical applications [15,16]. Luckily, the presence of free hydroxyl and amine groups in the chitosan backbone provide an opportunity to upgrade it with strongly hydrophilic functionalized molecules via simple chemical modification methods, which can not only improve the physical and chemical properties of chitosan but also expand its applications ranges [17,18].

As a member of the B-vitamins, α-lipoic acid is a safe, natural organosulfur compound with amphipathicity, derived from octanoic acid [19]. It has received increasing attention because of its pharmacological and medical value [20,21]. α-Lipoic acid exists in various parts of the body; both itself and its reduced form have the capacity to sustain the intrinsic cellular antioxidant defense system by mainly acting as biological antioxidants, metal-chelating agents, reducing agents of oxidized forms of other antioxidants such as vitamin C and E, and glutathione, and, finally, as modulators of some intracellular signaling pathways such as insulin and nuclear factor-κB [22,23]. Therefore, it can be regarded as an effective strategy to graft α-lipoic acid onto chitosan to improve the antioxidant ability of chitosan. The incorporation of α-lipoic acid can not only change the structural characterization, but also improve the physicochemical and biological properties of chitosan. Shen et al., (2020) reported the preparation of novel nanomicelles based on chitosan-graft-α-lipoic acid and these micelles showed excellent water dispersability, stability, and improved antioxidant activity of quercetin [24]. Luo et al., (2020) obtained the reduction-responsive copolymers based on chitosan–α-lipoic acid conjugates and they could be used as new copolymer carriers for the drug delivery of methotrexate [25]. However, the poor water solubility and the use of organic catalysts in the preparation process of chitosan–α-lipoic acid conjugates could inevitably limit their further extensive application.

In this work, the novel water-soluble chitosan derivatives were synthesized using HACC and α-lipoic acid as the reaction precursors in an aqueous medium via the ion exchange method. The chemical structures of the obtained chitosan derivatives were characterized by FTIR, ^1^H NMR, and ^13^C NMR. The quantitative data on degrees of substitution of the derivatives were calculated by the integral areas in ^1^H NMR spectra. In addition, their reducing power and scavenging abilities against hydroxyl radicals and DPPH radicals were also examined.

## 2. Results and Discussion

### 2.1. Chemical Synthesis and Characterization

**Scheme 1:**
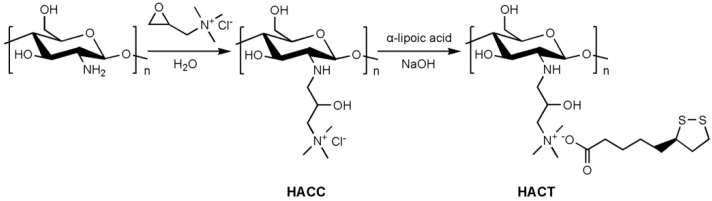
Synthetic routes for chitosan derivatives.

HACC is one of the most important cationic chitosan derivatives with great water solubility due to the presence of quaternary ammonium salt moieties [26,27,28]. In this paper, as shown in Scheme 1, HACC was obtained in deionized water without any organic solvent using chitosan and glycidyl trimethyl ammonium chloride as starting materials, by the nudeophilic ring-opening reaction between an epoxide ring and primary amine groups. The end products, HACTs with four different degrees of substitution, were prepared using HACC and thioctate, which was the salt form of α-lipoic acid in sodium hydroxide solution, as the reaction precursors at different feeding ratios by ion exchange in an aqueous system.

**Figure 1:**
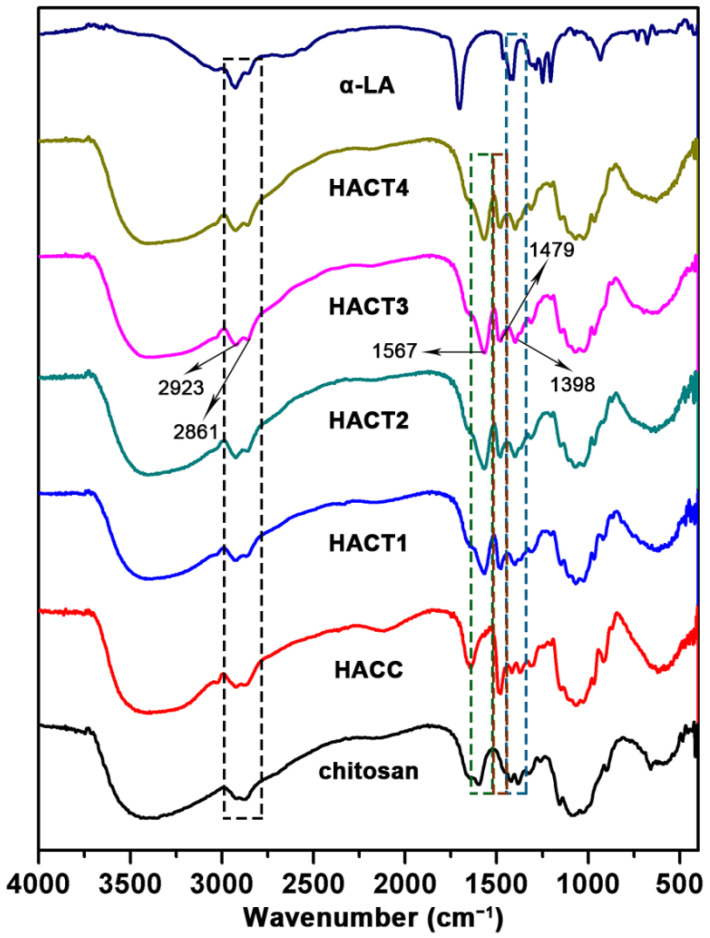
FTIR spectra of chitosan and chitosan derivatives.

The chemical structures of pristine chitosan and chitosan derivatives were analyzed by FTIR spectra (Figure 1). The spectrum of pristine chitosan showed its characteristic bands, namely: a broad band at 3410 cm^−1^ due to the free O–H and N–H symmetric stretching vibration and the intra- and inter-molecular hydrogen bonds; weak bands at 2920–2873 cm^−1^ assigned to the C–H stretch vibrations; characteristic peaks at 1648 and 1598 cm^−1^ ascribed to the C=O stretching (amide I) and N–H stretching (amide II), respectively; and the absorption peak at 1081 cm^−1^ assigned to the C–O stretching vibrations [29,30]. After the chitosan was modified with glycidyl trimethyl ammonium chloride, the new significant peak appears at 1479 cm^−1^, attributed to the C–H bending vibrations, which suggests the generation of an *N*,*N*,*N*-trimethyl quaternary ammonium group [31]. Proof of the successful introduction of α-lipoic acid onto the chitosan backbone was extracted from the FT-IR spectra and a significant new peak at 1567 cm^−1^ is observed, assigned to a carbonyl group (C=O) in a carboxylic acid anion of thioctate [32,33]. Meanwhile, increasing intensity of the adsorption bands at 2923–2861, and 1398 cm^−1^, ascribed to the C–H stretch vibrations of the aliphatic group, are observed in the spectra of all HACTs [34], compared with the pristine chitosan and HACC, also indicating that HACTs have been synthesized successfully.

**Figure 2:**
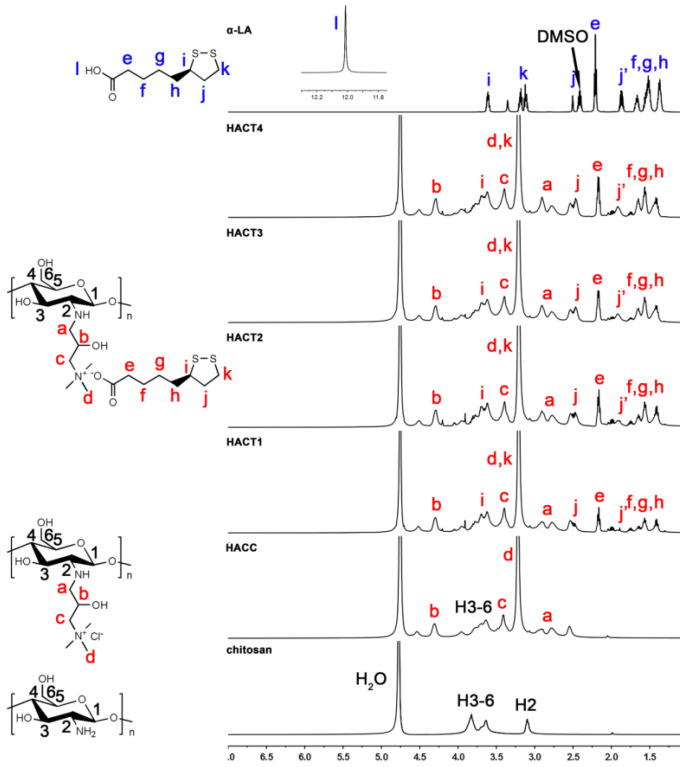
^1^H NMR spectra of chitosan and chitosan derivatives.

The chemical structures of the parent chitosan and the new derivatives were confirmed by ^1^H NMR, as shown in Figure 2. The ^1^H NMR spectrum of chitosan has been examined in previous studies and the signals can be interpreted as: 3.10 ppm (H2), 3.64–3.82 ppm (H3–H6) [35,36]. In the spectrum of HACC, additional signals at 4.31 (assigned as b), 3.41 (assigned as c), 3.22 (assigned as d), and 2.77–2.90 (assigned as a) ppm can be assigned to the protons of the newly formed hydroxypropyltrimethyl ammonium moieties, respectively [26,37]. There were new peaks in the range of 1.41–2.50 (assigned as e, f, g, h, j), and 3.69 ppm (assigned as i) in the spectra of HACTs when compared to the spectrum of HACC, which could be assigned to the methylene and methine protons of long chain alkyl, originating from the grafting of the α-lipoic acid onto the chitosan backbone [24]. The new chemical shifts observed for HACTs confirmed the successful introduction of α-lipoic acid onto the chitosan backbone via the ion exchange process. From the ^1^H NMR data, the DS values of the chitosan derivatives were calculated (Table 1) and the DS value of the quaternary ammonium salts was determined to be 1.55. By changing the feeding ratio of thioctate to aminoglucose units, the DS values of thioctate were from 51 to 86 per 100 anhydroglucose units of chitosan, respectively. These values indicated that the chitosan derivatives prepared in this paper, obtained at a higher feeding ratio of thioctate to aminoglucose units, had higher DS values.

**Figure 3:**
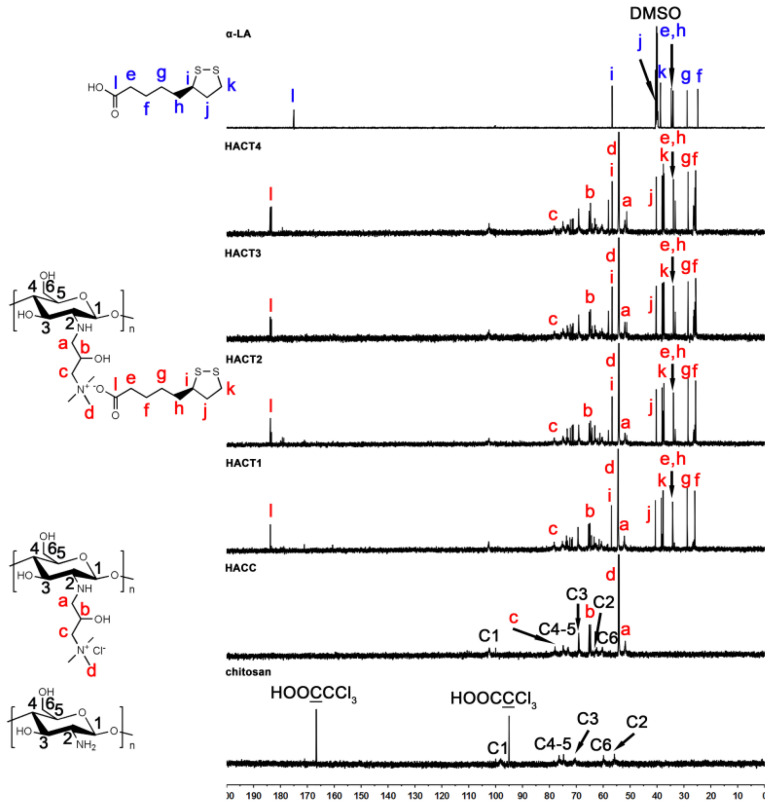
^13^C NMR spectra of chitosan and chitosan derivatives.

^13^C NMR spectroscopy was used for further investigation of the structures of the prepared chitosan derivatives. As shown in Figure 3, on the one hand, the peaks ascribed to the carbons in the chitosan backbone at 98.1 ppm (C-1), 55.8 ppm (C-2), 70.4 ppm (C-3), 76.4 ppm (C-4), 74.7 ppm (C-5), and 59.9 ppm (C-6) are evidently observed [38]. Additionally, the new ^13^C NMR signals at 51.8 ppm (a), 64.8 ppm (b), 77.9 ppm (c) and 54.3 ppm (d) for HACC are associated with the carbons of hydroxypropyltrimethyl ammonium moieties [26,28]. Other new signals at approximately 183.3 ppm (l), 56.7 ppm (i), 40.4 ppm (j), 37.7 ppm (k), 33.3–34.0 ppm (e, h), 28.5 ppm (g), and 26.0 ppm (f) are present in HACTs when compared with HACC, which were correlated to the carbons of thioctate [39].

The original FTIR, ^1^H NMR, and ^13^C NMR recorded spectra of raw materials and products were also provided in Supplementary Information (Figures S1–S7). The analytical results of the FTIR, ^1^H NMR, and ^13^C NMR spectra provided support for the success synthesis of HACTs.

### 2.2. Water Solubility

**Figure 4:**
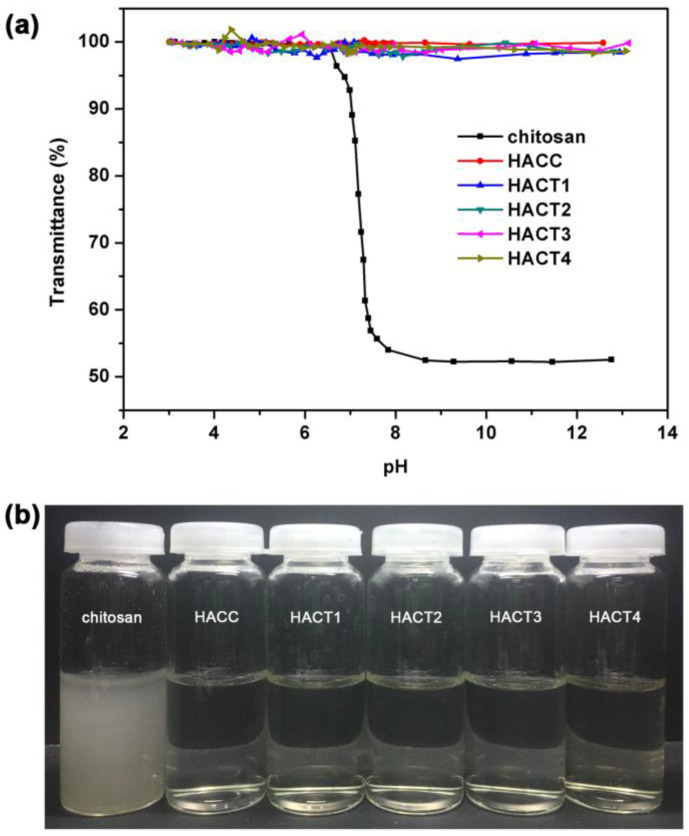
(**a**) pH dependence of water solubility and (**b**) optical photograph of chitosan and chitosan derivatives.

It was widely known that pristine chitosan and α-lipoic acid have poor solubility in neutral water. The effect of pH on water solubility of the chitosan derivatives prepared in this work was shown in Figure 4a. The fluctuation of pH directly affected the water solubility of pristine chitosan. At a low pH (pH < 6.0), chitosan had good solubility with the transmittance above 99%. When the pH was gradually increased from 7.0 to 13.0, the transmittance of the chitosan solutions decreased rapidly and became cloudy. In contrast, good solubilities of HACC and HACTs, without being affected by pH, in a wide range from 3.0 to 13.0, were observed. Meanwhile, the water solubility of chitosan, HACC, and HACTs were investigated, and the results were summarized in Table 1 and Figure 4b, which showed that HACC and HACTs could be completely dissolved in deionized water at a concentration of 50 mg/mL. These results indicated that the introduction of an *N*,*N*,*N*-trimethyl quaternary ammonium moiety into the chitosan backbone could greatly enhance water solubility and a combination of quaternization and an ion exchange method provided a highly efficient tool to prepare water-soluble chitosan derivatives with fat-soluble functional organic acids.

### 2.3. Antioxidant Activity

Three methods, namely, reducing power, DPPH, and hydroxyl radical scavenging activities, were used to evaluate the antioxidant activities of the prepared HACTs in vitro.

#### 2.3.1. Reducing Power

**Figure 5:**
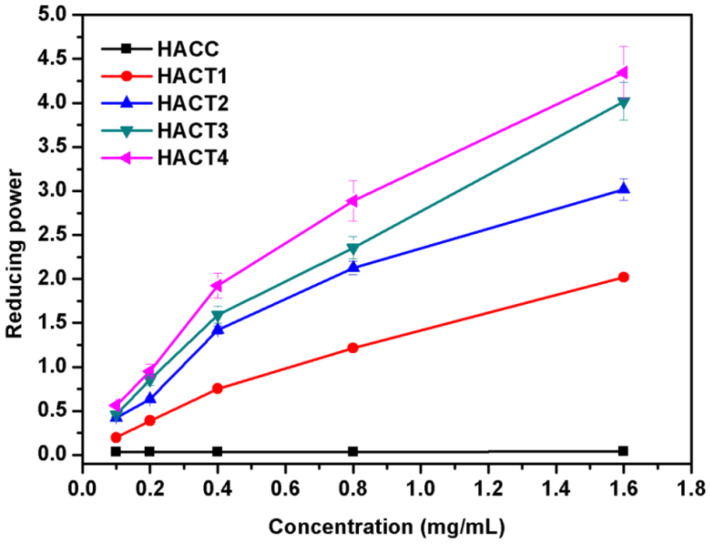
Reducing power of chitosan derivatives.

The reducing powers of HACTs as a function of the test concentration are presented in Figure 5. No reducing power was detected for HACC, while HACTs exhibited obvious increase in the absorbance values at 700 nm, which meant stronger reducing power. The results showed that as the concentration increases from 0.10 to 1.60 mg/mL, the reducing powers of HACTs showed a significant dose–effect relationship, and the reducing power reached the maximum when the test concentration was 1.60 mg/mL. At the maximum test concentration of 1.60 mg/mL, the absorbance values at 700 nm of HACTs under the test conditions are 2.020 ± 0.034, 3.018 ± 0.123, 4.017 ± 0.215, and 4.346 ± 0.296, respectively, as the DS values of thioctate increase, which indicated that the content of disulfide bonds had a direct positive influence on the reducing power.

#### 2.3.2. DPPH Radical Scavenging Activity

**Figure 6:**
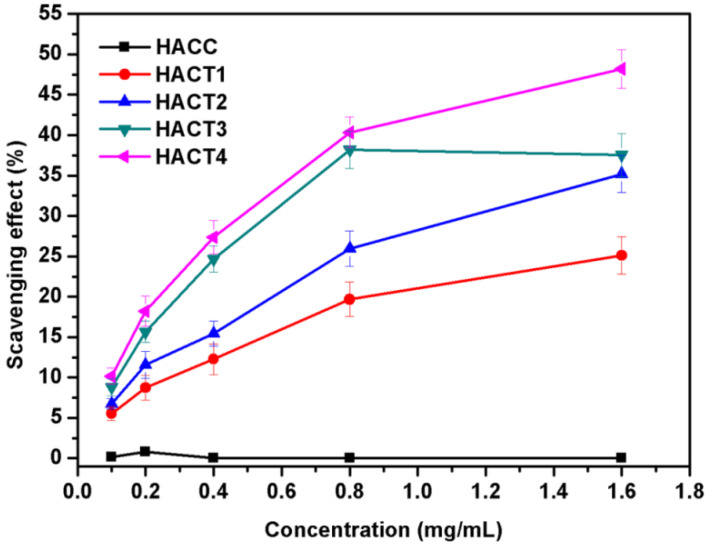
DPPH radical scavenging activity of chitosan derivatives.

The purple DPPH radicals can turn into a yellow stable compound when reacted with antioxidants and the DPPH radical scavenging assay is one of the most widely-used methods for the measurement of a free radical’s ability to scavenge antioxidants, due to their simplicity, rapidity, and sensitivity [8]. As shown in Figure 6, the test concentration of samples ranges from 0.10 to 1.60 mg/mL, the scavenging rate of HACC is below 1%. The prepared HACTs showed diverse abilities in scavenging DPPH radicals, and all of their abilities were stronger than that of HACC, which might result from the incorporation of α-lipoic acid. Besides, the scavenging activities of HACTs against DPPH radicals showed a concentration-dependent manner, and HACTs at maximum test concentration of 1.60 mg/mL exhibited the highest scavenging effect against DPPH radicals. Meanwhile, as the DS values of thioctate increased, the scavenging activity against DPPH radicals of HACTs also increased.

#### 2.3.3. Hydroxyl Radical Scavenging Activity

**Figure 7:**
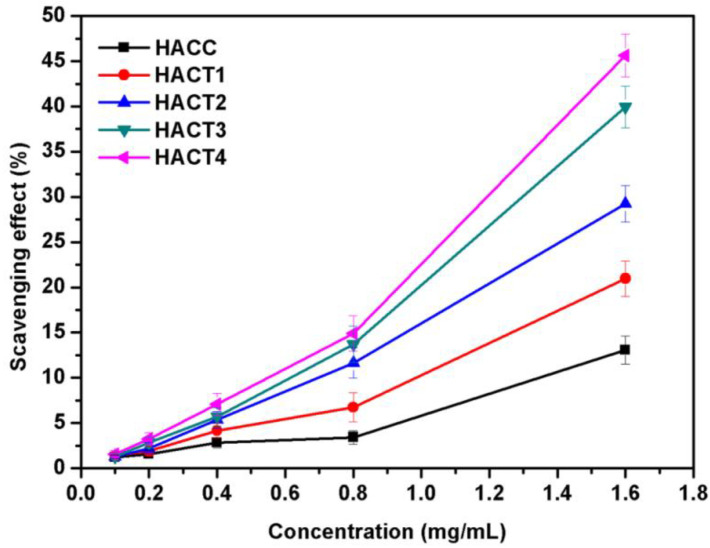
Hydroxyl radical scavenging activity of chitosan derivatives.

As highly reactive and harmful oxidants to living organisms, hydroxyl radicals can cause serious damage to DNA, lipids, and proteins [40]. Therefore, it is essential to remove hydroxyl radicals for protecting life systems. As can be seen from Figure 7, all samples show the scavenging activity against hydroxyl radicals. When the test concentration was increased from 0.10 to 1.60 mg/mL, the dose–effect relationship was obvious and the results indicated that, upon increasing the concentration to 1.60 mg/mL, the scavenging rate reached the maximum. The hydroxyl radical scavenging activity of HACC was only about 10%, while the hydroxyl radical scavenging activities of HACTs were significantly increased from 20% to 45% after grafting alpha lipoic acid. The increased radical scavenging activity might be attributed to the property of alpha lipoic acid as an effective bio-antioxidant. In addition, it was noticed that an increase in DS values (from 0.51 to 0.86) of thioctate in the HACTs led to a significant increase in the scavenging activity against hydroxyl radicals. The result of the antioxidant capacity evaluated by the scavenging assay against hydroxyl radicals was very consistent with those determined by the reducing power assay and the scavenging assay against DPPH radicals.

## 3. Materials and Methods

### 3.1. Materials

Chitosan (molecular weight: 575 KDa, deacetylated degree: 95%) was supplied by Zhejiang Golden-Shell Pharmaceutical Co., Ltd. (Yuhuan, China). Glycidyl trimethyl ammonium chloride, (R)-(+)-lipoic acid, and sodium hydroxide were purchased from the Sinopharm Chemical Reagent Co., Ltd. (Shanghai, China). Other chemicals of analytical-reagent-grade were used as received.

### 3.2. Preparation of Chitosan Derivatives

#### 3.2.1. Synthesis of Hydroxypropyltrimethyl Ammonium Chloride Chitosan (HACC)

Chitosan (3.22 g, 20 mmol of glucosamine), glycidyl trimethyl ammonium chloride (9.58 g, 60 mmol), and deionized water (200 mL) were added to a 500 mL round-bottomed flask equipped with a mechanical agitator and a condenser. The mixture was warmed to 80 °C and vigorously stirred at reflux for 48 h. The reaction mixture was then cooled down to room temperature. After being dialyzed against deionized water for 2 days to remove the probable remaining reagents, the undissolved precipitate was discarded by centrifugation and HACC was obtained by lyophilization of the supernatant overnight in a vacuum. Yield: 49%. FTIR (KBr): 3405, 2921, 2881, 1647, 1479, and 1068 cm^−1^. ^1^H NMR (D_2_O): *δ* 2.77, 2.90, 3.22, 3.41, 3.64, 3.96, 4.31, and 4.53 ppm. ^13^C NMR (D_2_O): *δ* 51.8, 54.3, 60.3, 62.4, 64.8, 69.2, 73.0, 74.9, 77.9, and 102.3.

#### 3.2.2. Synthesis of Hydroxypropyltrimethyl Ammonium Chitosan Derivatives Bearing Thioctate (HACTs)

Hydroxypropyltrimethyl ammonium chitosan derivatives bearing thioctate, with various degrees of substitution of thioctate, were synthesized according to the following procedure: HACC (1.50 g) was dissolved completely in deionized water (100 mL) and added to a 250 mL round-bottomed flask equipped with a mechanical agitator. The thioctate aqueous solution was prepared by dissolving α-lipoic acid (molar ratios of α-lipoic acid to HACC were 1:1, 1:2, 1:3, and 1:5, respectively), in an equivalent amount of 40% (*w*/*v*) NaOH aqueous solution under constant stirring for 0.5 h at 25 °C. Then, the different dosages of the above mixture were added slowly to the HACC aqueous solution, respectively, followed by stirring vigorously for 12 h at 25 °C. After that, the reaction solution was purified by dialysis (molecular weight cutoff: 500, MD77, USA) in deionized water for 2 days. Finally, the purified HACTs were obtained by freeze-drying overnight in a vacuum. The four different products were named as HACT1, HACT2, HACT3, and HACT4, respectively.

HACT1: FTIR (KBr): 3387, 2923, 2867, 1567, 1477, 1399, 1068 cm^−1^. ^1^H NMR (D_2_O): *δ* 1.41, 1.57, 1.75, 1.89, 2.16, 2.50, 2.78, 2.91, 3.22, 3.40, 3.62, 3.69, 4.30, 4.52 ppm. ^13^C NMR (D_2_O): *δ* 25.7, 28.5, 34.0, 37.5, 38.2, 40.4, 52.0, 54.3, 56.8, 60.5, 62.4, 64.8, 69.2, 73.4, 74.9, 78.1, 102.3, and 183.6.

HACT2: FTIR (KBr): 3400, 2923, 2861, 1568, 1479, 1400, 1072 cm^−1^. ^1^H NMR (D_2_O): *δ* 1.41, 1.57, 1.65, 1.91, 2.16, 2.47, 2.78, 2.91, 3.22, 3.39, 3.61, 3.69, 4.29, 4.51 ppm. ^13^C NMR (D_2_O): *δ* 25.7, 28.5, 34.0, 37.5, 38.2, 40.4, 51.9, 54.3, 56.7, 60.3, 63.1, 64.8, 69.3, 74.9, 78.1, 102.3, and 183.6.

HACT3: FTIR (KBr): 3384, 2923, 2861, 1567, 1479, 1398, 1070 cm^−1^. ^1^H NMR (D_2_O): *δ* 1.42, 1.57, 1.65, 1.92, 2.18, 2.47, 2.78, 2.91, 3.22, 3.40, 3.61, 3.69, 4.29, 4.51 ppm. ^13^C NMR (D_2_O): *δ* 25.7, 28.5, 34.0, 37.5, 38.1, 40.4, 52.0, 54.3, 56.7, 60.6, 62.7, 69.0, 73.1, 75.0, 78.4, 102.6, and 183.3.

HACT4: FTIR (KBr): 3398, 2925, 2857, 1568, 1479, 1398, 1068 cm^−1^. ^1^H NMR (D_2_O): *δ* 1.42, 1.57, 1.65, 1.91, 2.17, 2.47, 2.78, 2.90, 3.22, 3.39, 3.61, 3.69, 4.29, 4.51 ppm. ^13^C NMR (D_2_O): *δ* 25.7, 28.5, 34.0, 37.5, 38.2, 40.4, 52.0, 54.3, 56.7, 60.3, 62.9, 64.8, 69.3, 73.3, 75.0, 78.1, 102.3, and 183.3.

### 3.3. Characterization of Chitosan Derivatives

#### 3.3.1. Fourier Transform Infrared (FTIR) Spectroscopy

The functional groups of the pristine chitosan, HACC, and HACTs were analyzed by FT-IR spectra. The spectra were recorded using Nicolet iS50 instrument (Thermo, Waltham, MA, USA) at 25 °C using the transmittance mode in the wavenumber range from 4000 to 400 cm^−1^ with a resolution of 4.0 cm^−1^. All the tested samples were made into tablets by mixing with analytical grade KBr for the observations. The scan was performed against a blank KBr pellet background with accumulation of 32 scans.

#### 3.3.2. Nuclear Magnetic Resonance (^1^H NMR and ^13^C NMR) Spectroscopy

The chemical structures of the pristine chitosan, HACC, and HACTs were further confirmed by ^1^H NMR and ^13^C NMR spectra. These spectra were carried out on an AVANCE IIITM 500 Spectrometer (Bruker, Zurich, Switzerland) under a static magnetic field of 500 MHz and 125 MHz at 25 °C. Pristine chitosan was dissolved in 2% trichloroacetic acid deuterium oxide (D_2_O) mixtures, and the chitosan derivatives were dissolved in D_2_O, but α-lipoic acid was dissolved in (CD_3_)_2_SO (DMSO-*d*6). Chemical shifts were depicted as *δ* (ppm) and reported relative to the solvent peaks. Meanwhile, the degrees of substitution (DS) of the prepared chitosan derivatives were obtained by the integral areas in ^1^H NMR spectra and calculated by the following Equations (1) and (2).

DS_1_ (%) = ((5 × ∫ H_b_)/∫ H_3__–6_) × 100 (1)

DS_2_ (%) = (∫ H_e_/(2 × ∫ H_b_)) × DS_1_ × 100 (2)

where DS_1_ is the degree of substitution of hydroxypropyltrimethyl ammonium in HACC, DS_2_ is the degree of substitution of thioctate in HACTs, ∫ H_3–6_ is the integral area of H_3_–H_6_ (*δ* = 3.53–4.12 ppm) in the glucosamine unit, ∫ H_b_ is integral area of H_b_ (singlet at 4.30 ppm) in hydroxypropyltrimethyl ammonium salt and ∫ H_e_ is integral area of H_e_ (multiplet at 2.17 ppm) in the thioctate group.

### 3.4. Water Solubility

The method of turbidimetric titration was used to determine the solubility of the chitosan derivatives at different pH ranges. The pristine chitosan and the chitosan derivatives (20 mg) were dissolved in a 1% acetic acid solution (20 mL) at 25 °C, respectively. Then the pH of solutions was gradually regulated by a 1 M NaOH solution and its value was obtained using an FE28-Standard pH meter (METTLER TOLEDO, Uster, Switzerland), followed by the transmittance at λ = 600 nm, recorded by a Lambda 265 UV–visible spectrometer (PerkinElmer, Waltham, MA, USA). Furthermore, test samples (500 mg) were stirred in deionized water (10 mL) at 25 °C for 12 h, respectively, and the insoluble parts were then collected by centrifugation, washed with absolute ethanol, freeze-dried, and weighed. The water solubility was calculated according to the following Equation (3):

Water solubility (mg/mL) = (500 − W)/10 (3)

where W (mg) is the weight of undissolved parts.

### 3.5. Evaluation of Antioxidant Activity In Vitro

#### 3.5.1. Reducing Power Assay

The capacity of the prepared chitosan derivatives to reduce iron (III) was measured according to the reported method [41]. A total of 1.0 mL of the sample solutions with various concentrations (0.60, 1.20, 2.40, 4.80, and 9.60 mg/mL) were mixed with 1.0 mL of potassium ferricyanide (K_3_[Fe(CN)_6_], 1.0% (*w*/*v*)) and incubated at 50 °C for 20 min. The reaction was stopped by the addition of 1.0 mL of trichloroacetic acid solution (CCl_3_COOH, 10% (*w*/*v*)), followed by centrifugation at 3000 rpm for 5 min. A total of 1.5 mL of each supernatant was mixed with 0.3 mL of ferric chloride solution (FeCl_3_, 0.1% (*w*/*v*)) and 1.2 mL of distilled water, and then incubated at room temperature for 10 min. Finally, the absorbance of the resultant solution was measured at 700 nm. Higher absorbance of the reaction mixture showed a stronger reducing power. The experiments were carried out in triplicate.

#### 3.5.2. DPPH Radical Scavenging Assay

The ability of the chitosan derivatives to scavenge DPPH radicals was measured according to the previous literature [38]. Briefly, 1.0 mL of the sample solutions at various concentrations (0.3 mg/mL, 0.6 mg/mL, 1.2 mg/mL, 2.4 mg/mL, and 4.8 mg/mL) were added into 2.0 mL of the DPPH solution (0.18 mM in absolute ethanol) and kept at room temperature in the dark for 20 min before measuring in a UV–vis spectrophotometer at 517 nm. Then, deionized water, rather than sample solution, was used as a blank, and absolute ethanol was used instead of the DPPH solution was used as the control. The calculation used to report on the scavenging of the DPPH radicals was given by Equation (4):

Scavenging effect (%) = [1 − (A_1_ − A_2_)/A_0_] × 100 (4)

where A_0_ is the absorbance of the blank group, A_1_ is the absorbance of the sample group, and A_2_ is the absorbance of the control group.

#### 3.5.3. Hydroxyl Radical Scavenging Assay

The hydroxyl radical scavenging activity was measured based on the generation of hydroxyl radicals from a Fenton reaction between ferrous ions and H_2_O_2,_ according to a previously described method [42]. A total of 1.0 mL of sample solutions with various concentrations (0.45, 0.90, 1.80, 3.60, and 7.20 mg/mL) were mixed with 3.5 mL of a sodium phosphate buffer (pH 7.4, 150 mM), which contained 0.5 mL of EDTA-Fe^2+^ (2.0 mM), 1.0 mL of safranine T (1.0 mM), and 1.0 mL of H_2_O_2_ (3%, (*v*/*v*)). The reaction solutions were incubated at 37 °C for 30 min, and the absorbance was read at 520 nm by a spectrophotometer. A deionized water displaced sample was used as a blank and the sodium phosphate buffer replaced H_2_O_2_ as the control for all assays. The scavenging activity against hydroxyl radicals was calculated using Equation (5):

Scavenging effect (%) = (A_1_ − A_2_)/(A_0_ − A_2_) × 100 (5)

where A_0_ is the absorbance of the control group, A_1_ is the absorbance of the test group, and A_2_ is the absorbance of the blank group.

### 3.6. Statistical Analysis

All antioxidant experiments in this study were done in triplicate and data were presented as the mean ± standard deviations. Statistical significance (*p* < 0.05) was determined by a one-way analysis of variance and graphing was dealt with using Origin 8.0 (Northampton, MA, USA).

## 4. Conclusions

In this study, the preparation of hydroxypropyltrimethyl ammonium chitosan derivatives bearing thioctate, with four different degrees of substitution of thioctate, via an ion exchange method, was described. FTIR, ^1^H NMR, and ^13^C NMR spectroscopy confirmed the successful synthesis of the prepared chitosan derivatives, and the degrees of substitution of thioctate were calculated by the integral areas in the ^1^H NMR spectra. The antioxidant activity evaluated by the reducing power, DPPH, and hydroxyl radical scavenging activity assays showed that, compared with HACC, the final products HACTs exhibited a stronger radical scavenging capacity and reducing power, which further enhanced as the test concentrations and the degrees of substitution of thioctate increased. The increased antioxidant property of HACTs might be attributed to the property of the disulfide bond to act as an electron donor. The results demonstrated that the hydroxypropyltrimethyl ammonium chitosan derivatives bearing thioctate could have great potential application as antioxidant agents.

## Figures and Tables

**Table 1:** The degrees of substitution and water solubility of chitosan derivatives.

Compound	Chitosan	HACC	HACT1	HACT2	HACT3	HACT4
Degree of Substitution	0.95 ^a^	1.55 ^b^	0.51 ^c^	0.68 ^c^	0.80 ^c^	0.86 ^c^
Water Solubility (mg/mL)	/ *	≥50	≥50	≥50	≥50	≥50

^a^ Deacetylated degree of chitosan. ^b^ Degree of substitution of quaternary ammonium salts in HACC. ^c^ Degree of substitution of thioctate in HACTs. * Symbol ‘/’ represented insoluble.

## Data Availability

Not applicable.

## Supplementary Materials

The following supporting information can be downloaded at: https://www.mdpi.com/article/10.3390/molecules27092682/s1, Figure S1: FTIR, ^1^H NMR, and ^13^C NMR spectra of chitosan; Figure S2: FTIR, ^1^H NMR, and ^13^C NMR spectra of HACC; Figure S3: FTIR, ^1^H NMR, and ^13^C NMR spectra of HACT1; Figure S4: FTIR, ^1^H NMR, and ^13^C NMR spectra of HACT2; Figure S5: FTIR, ^1^H NMR, and ^13^C NMR spectra of HACT3; Figure S6: FTIR, ^1^H NMR, and ^13^C NMR spectra of HACT4; Figure S7: FTIR, ^1^H NMR, and ^13^C NMR spectra of α-lipoic acid.

## Sample Availability

Samples of the chitosan derivatives are available from the corresponding author upon reasonable request.
