# Supplementary material for: Synthesis of Hydroxypropyltrimethyl Ammonium Chitosan Derivatives Bearing Thioctate and the Potential for Antioxidant Application
Source: Molecules. 2022 Apr 21;27(9):2682. doi: 10.3390/molecules27092682 (PMC9101115; doi:10.3390/molecules27092682)
Supplement: Supplementary file 1 [file molecules-27-02682-s001.zip › molecules-1661984-supplementary.pdf]

Supplementary Materials

Synthesis of Hydroxypropyltrimethyl Ammonium Chitosan Derivatives Bearing Thioctate and the Potential for Antioxidant Application

Wenqiang Tan [1,2], Conghao Lin [3], Jingjing Zhang [1,2], Qing Li [1,2] and Zhanyong Guo [1,2,*]

1 Research and Development Center for Efficient Utilization of Coastal Bioresources, Yantai Institute of Coastal Zone Research, Chinese Academy of Sciences, Yantai 264003, China; wqtan@yic.ac.cn (W.T.); jingjingzhang@yic.ac.cn (J.Z.); qli@yic.ac.cn (Q.L.)

2 Center for Ocean Mega-Science, Chinese Academy of Sciences, 7 Nanhai Road, Qingdao 266071, China;

3 College of Life Sciences, Yantai University, Yantai 264005, China; conghaolin@s.ytu.edu.cn (C.L.)

* Correspondence: zyguo@yic.ac.cn

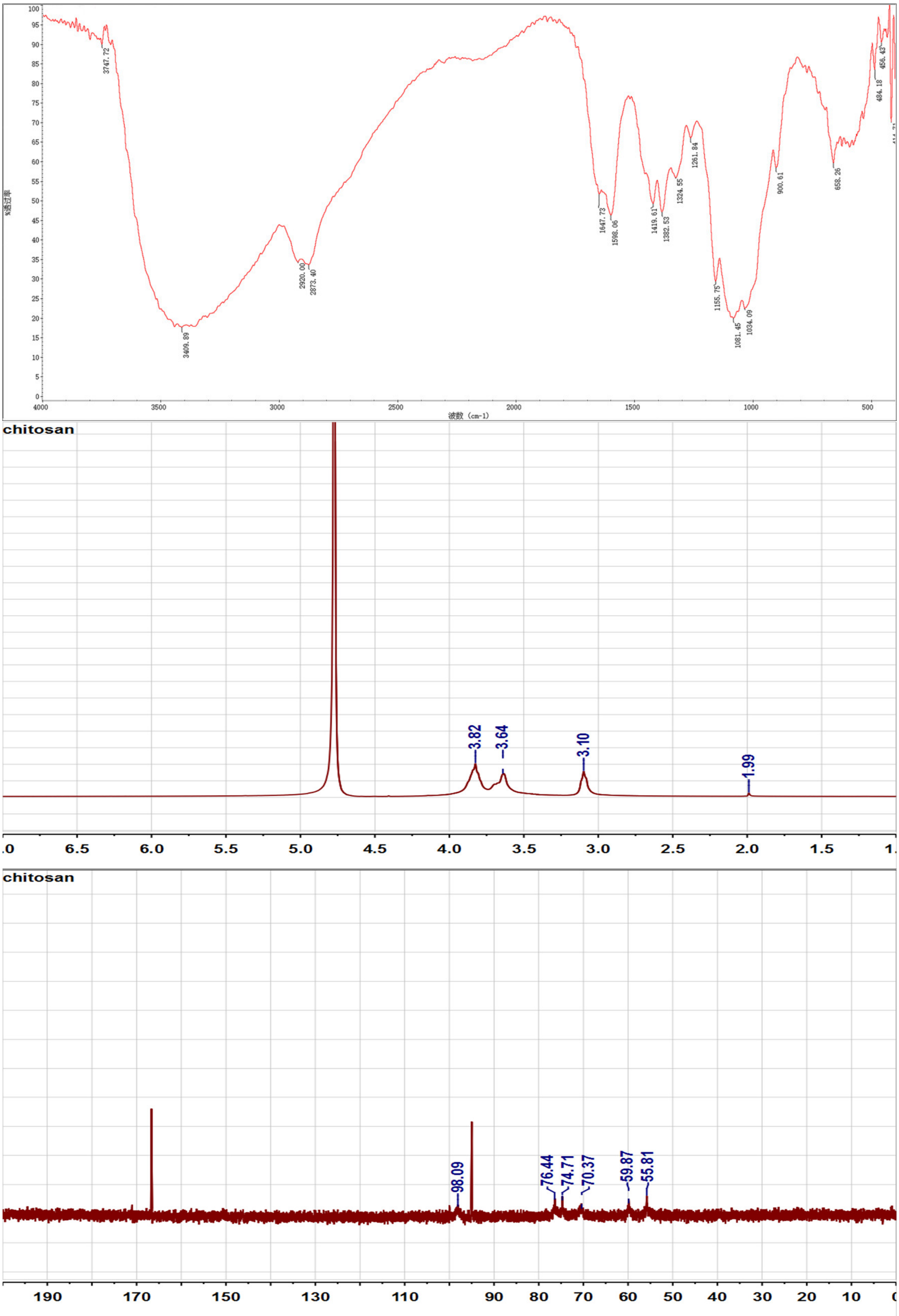

Figure S1. FTIR, ^{1}H NMR, and ^{13}C NMR spectra of chitosan.

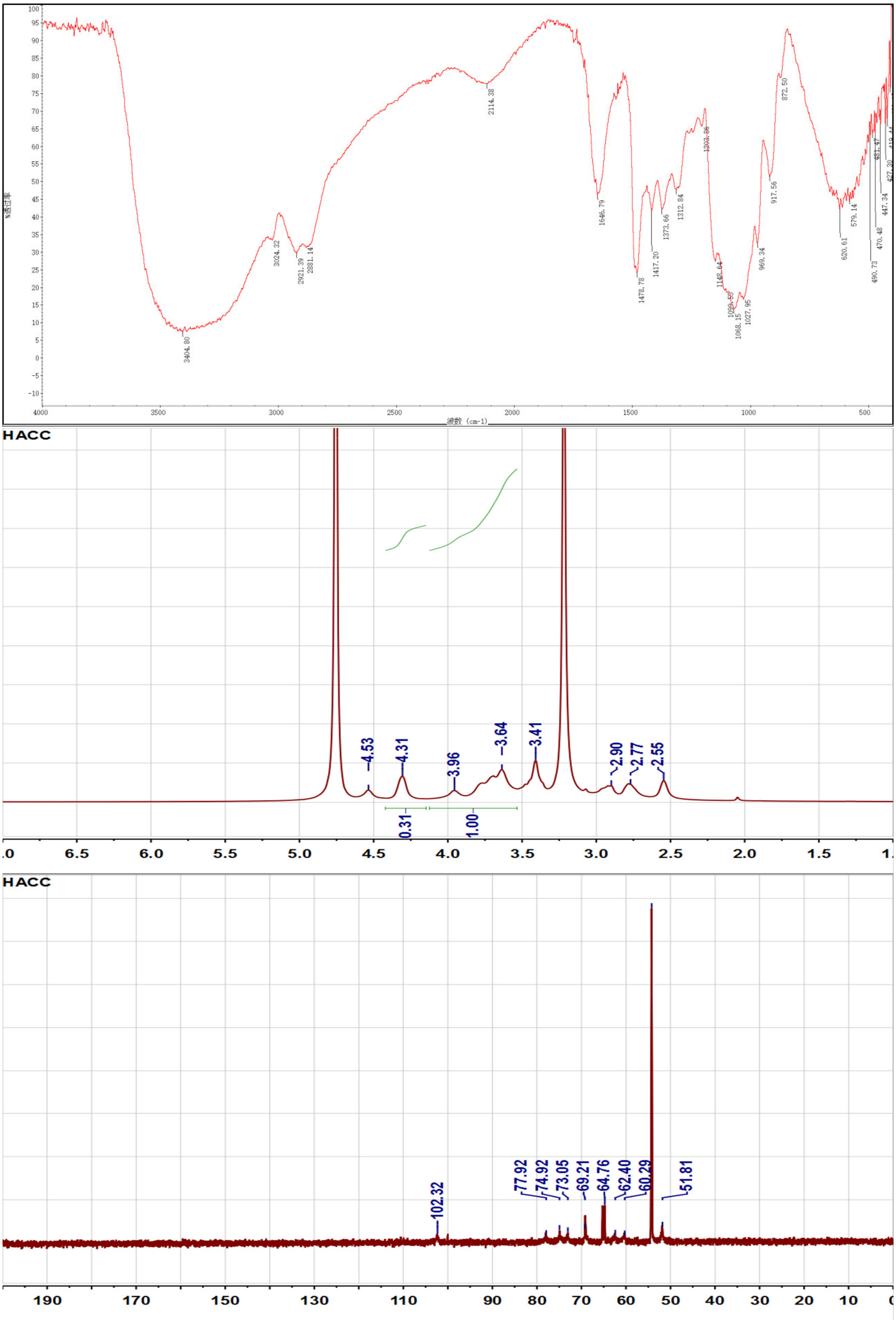

Figure S2. FTIR, ^{1}H NMR, and ^{13}C NMR spectra of HACC.

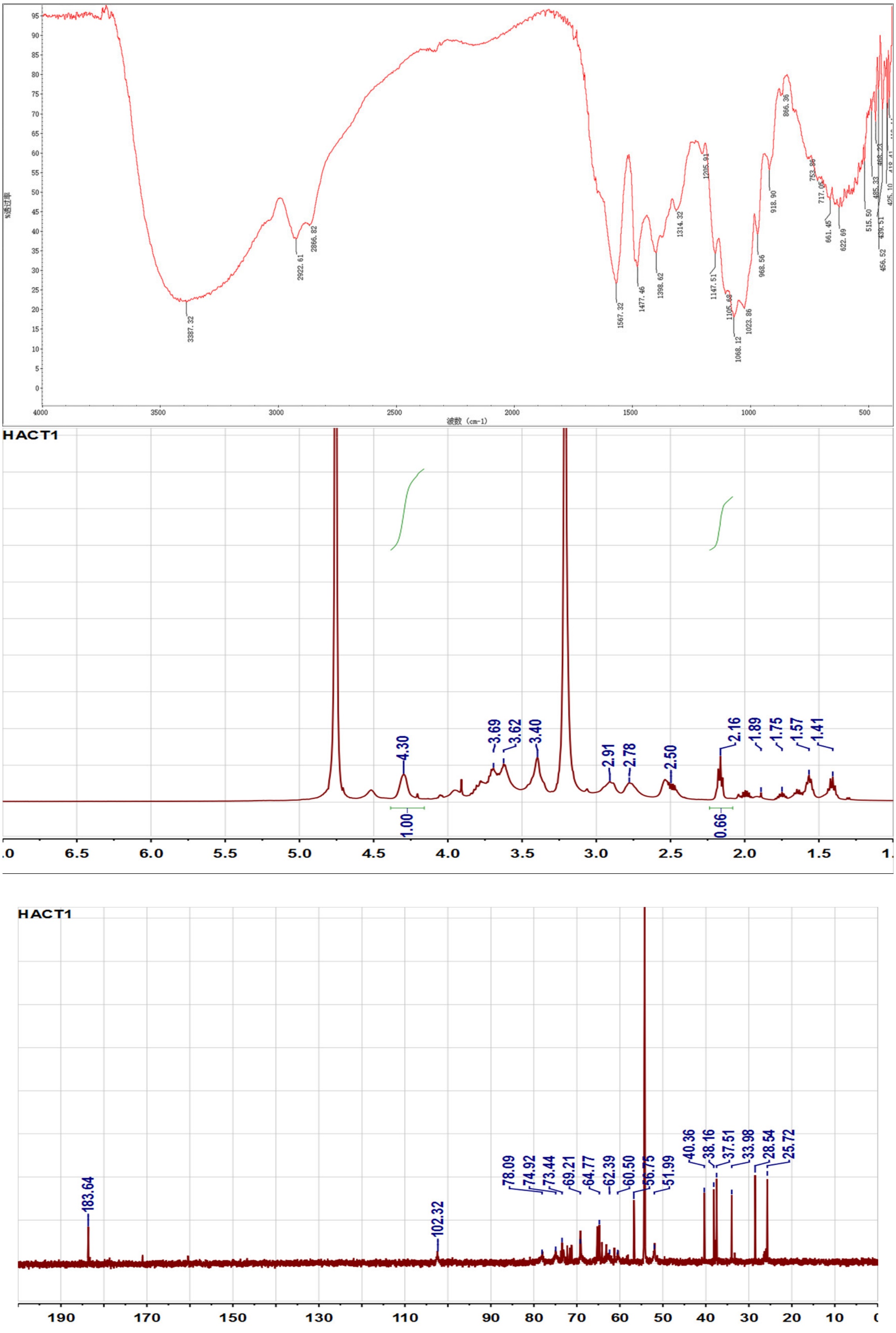

Figure S3. FTIR, ^{1}H NMR, and ^{13}C NMR spectra of HACT1.

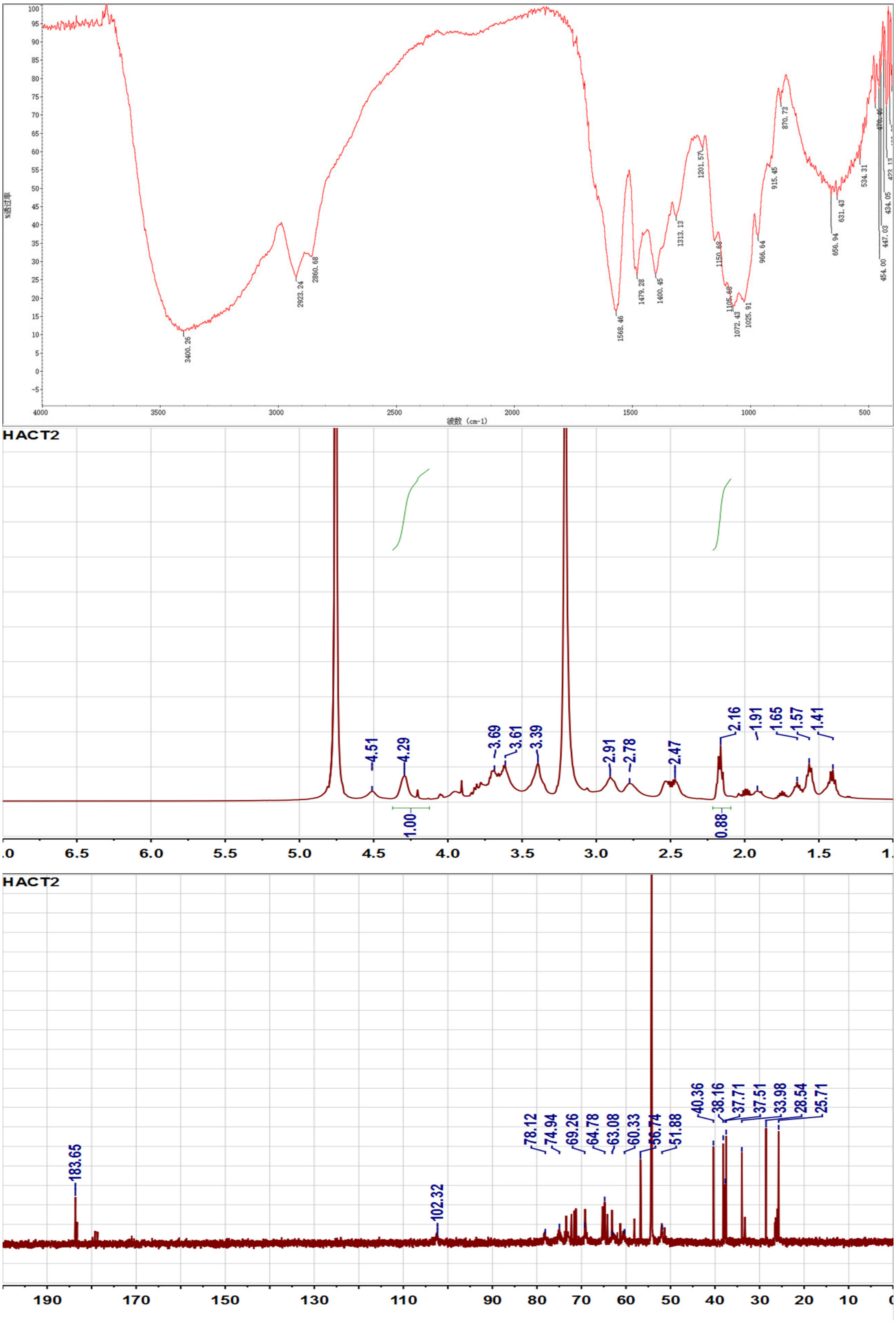

Figure S4. FTIR, ¹H NMR, and ¹³C NMR spectra of HACT2.

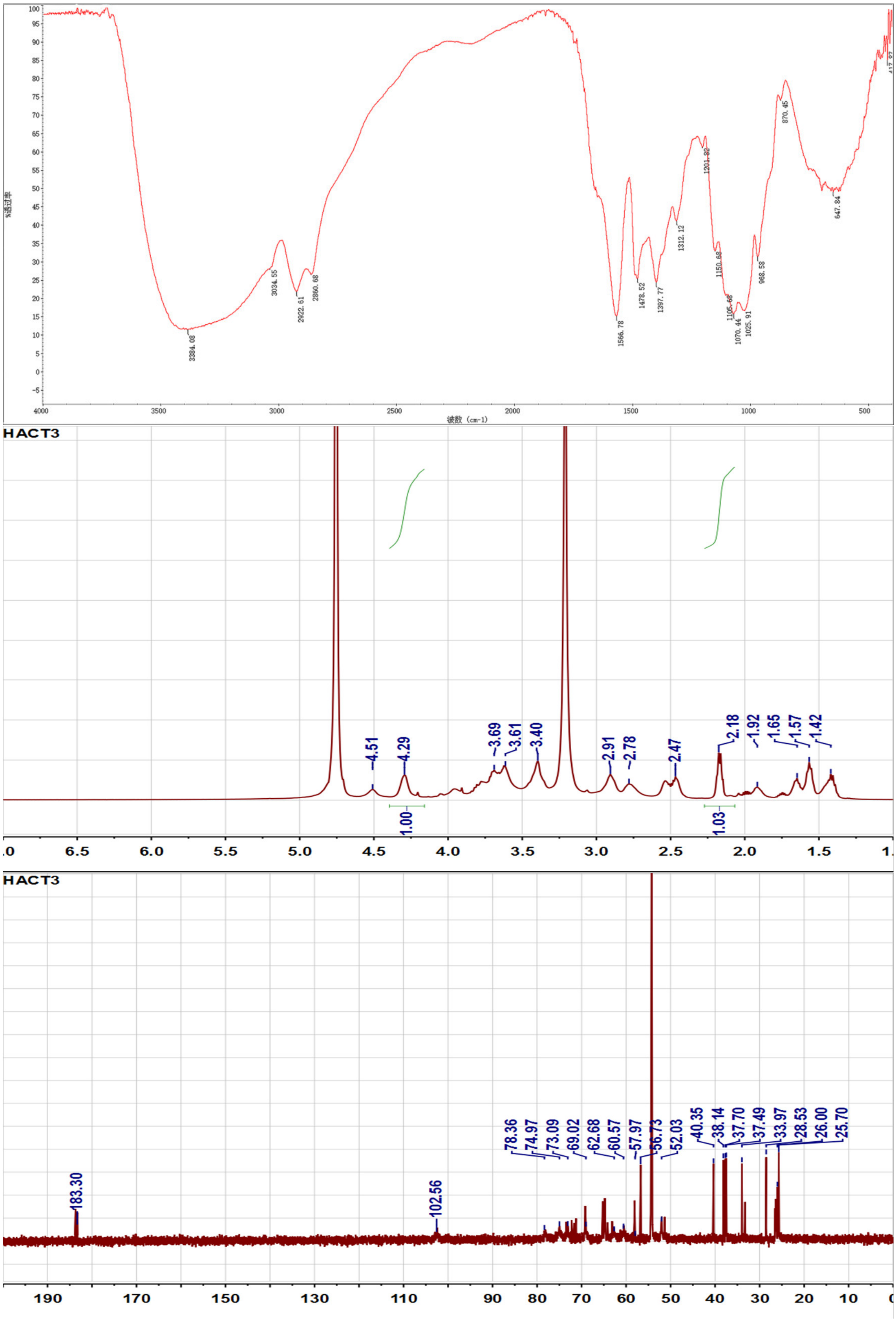

Figure S5. FTIR, ^{1}H NMR, and ^{13}C NMR spectra of HACT3.

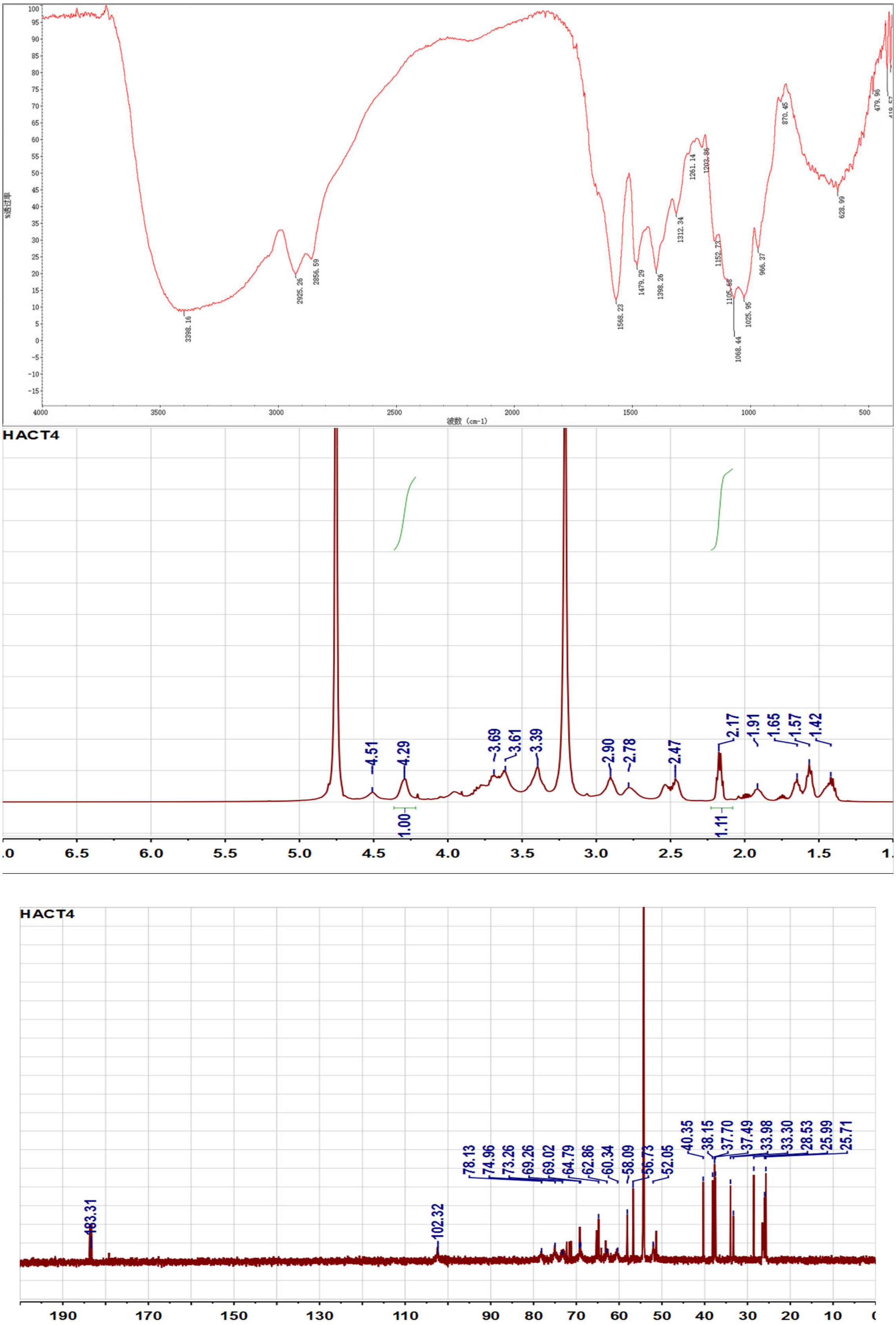

Figure S6. FTIR, ^{1}H NMR, and ^{13}C NMR spectra of HACT4.

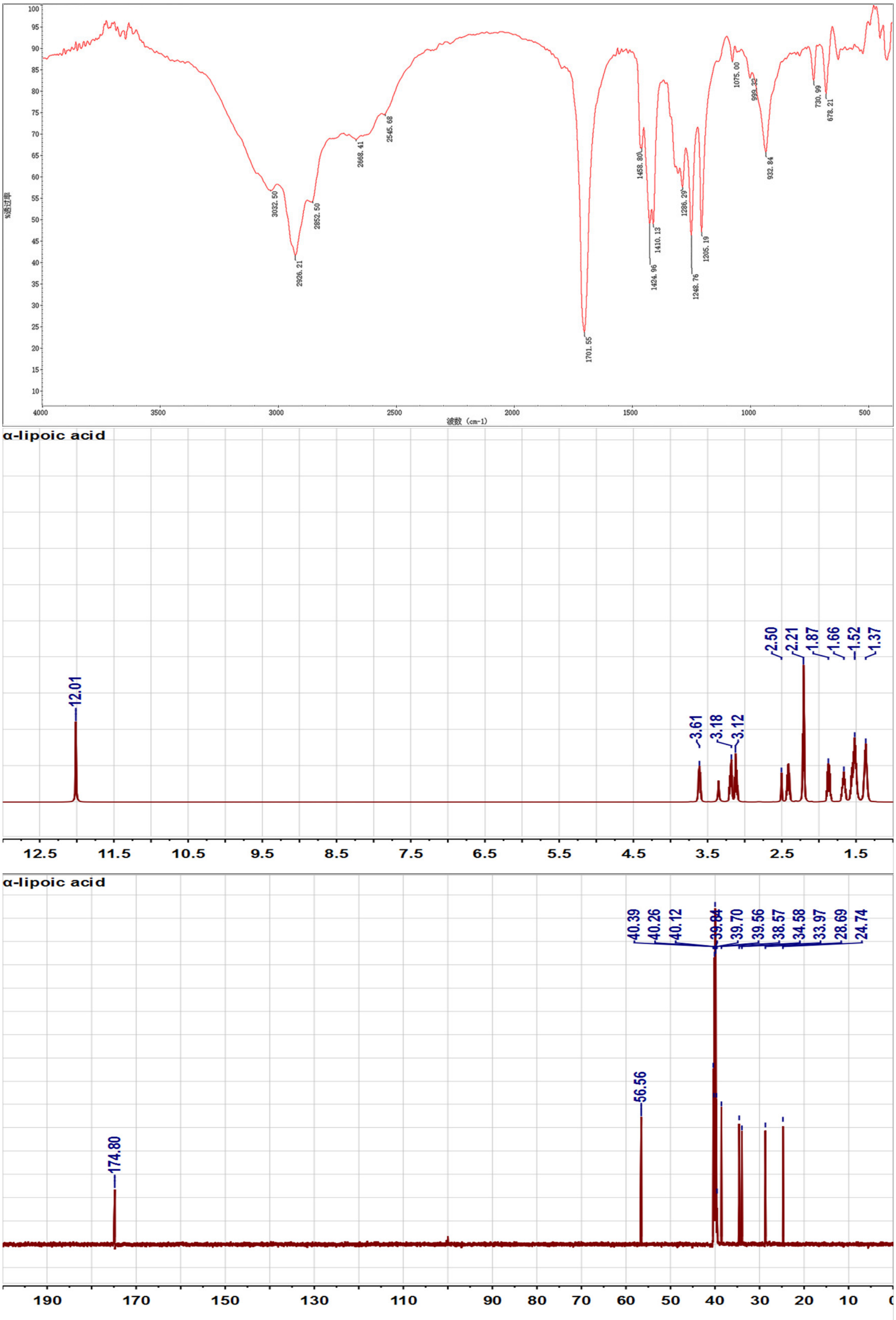

Figure S7. FTIR, ^{1}H NMR, and ^{13}C NMR spectra of α-lipoic acid.
